# 2352. The Dynamic Change in Humeral Immunity Against SARS-CoV-2 Omicron Subvariants in Patients with Breakthrough Infection: The Prospective Cohort Study

**DOI:** 10.1093/ofid/ofad500.1973

**Published:** 2023-11-27

**Authors:** Monprach Harnphadungkit, Opass Putcharoen, Supaporn Wacharapluesadee

**Affiliations:** Faculty of Medicine, Chulalongkorn University and King Chulalongkorn Memorial Hospital, Pathumwan, Krung Thep, Thailand; Division of Infectious Disease, Department of Medicine, Faculty of Medicine, Chulalongkorn University, Krungthep, Krung Thep, Thailand; Emerging infectious diseases clinical center, King Chulalongkorn Memorial Hospital, Pathumwan, Krung Thep, Thailand

## Abstract

**Background:**

The SARS-CoV-2 continued to emerge new variants. Omicron variants had become world predominance. There were increase in number of breakthrough infections. This study aimed to evaluated immunity after breakthrough infections in Thai patients, given high varieties of vaccination regimens.

**Methods:**

We conducted a cohort study at King Chulalongkorn Memorial Hospital (KCMH) and enrolled participants with breakthrough infections during April 2022 in outpatient setting. Serum sample was evaluated at baseline, 1-month, and 3-months post-infection. Surrogate virus neutralization test (sVNT), using cPASS GenScript^TM^, was used to evaluate the neutralizing capacity against SARS-CoV-2 wild-type and Omicron variants.

**Results:**

A total of 109 participants were enrolled, with a median age of 36 years (IQR: 28-45). Nine vaccination regimens were observed, with all but one participant receiving at least one dose of booster vaccination. The most common duration from last vaccination to enrollment was 3-4 months. SARS-CoV-2 Omicron BA.2 was the predominant strain (97.8%). At baseline, the highest sVNT was observed against wild-type, followed by Omicron BA.2 and Omicron BA.1. At 1-month post-infection, we observed a non-significant increase in sVNT against wild-type (p=0.11), but a significant increase in antibodies against Omicron BA.2 (p < 0.01) then stable at 3-month post-infection. Of the participants, 31 received a booster vaccine before the scheduled 3-month follow-up, and their sVNT levels at 3-month post-infection were similar to those who did not receive a booster. This study found no difference in post-infection sVNT level across previous vaccination regimens.

**Table 1:**
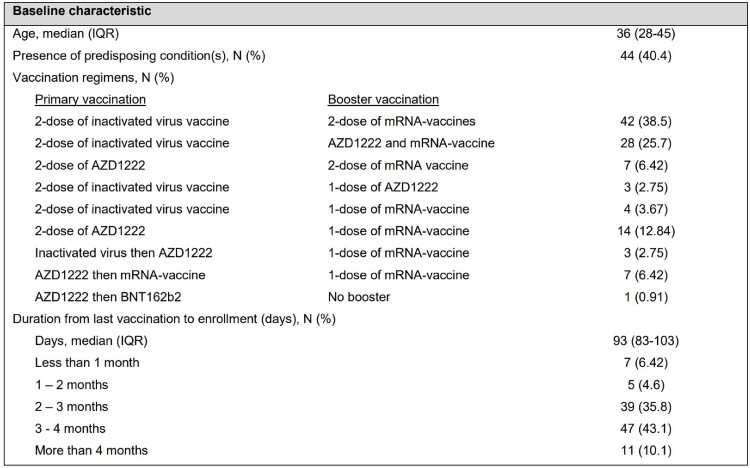
Baseline characteristics of participants

Table 1: Baseline characteristics of participants. Continuous data are presented as median and interquartile range (IQR). Categorical data are presented as number and percentage.

Baseline sVNT against SARS-CoV-2 wild type, Omicron BA.1, and Omicron BA.2

**Figure:**
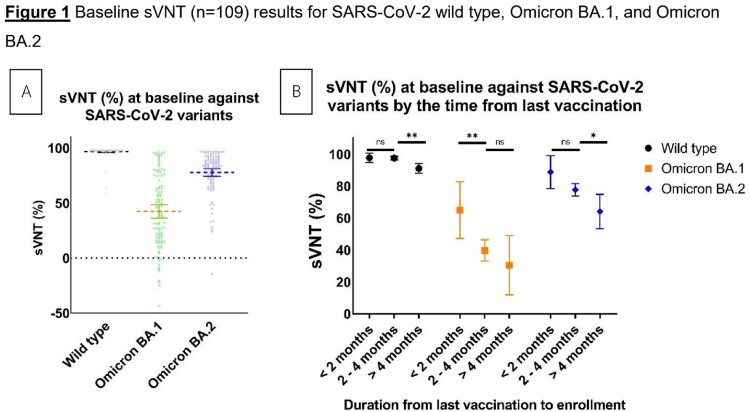

Figure 1A Baseline sVNT (%) results for SARS-CoV-2 wild type, Omicron BA.1, and Omicron BA.2. Figure 1B Baseline sVNT (%) results for SARS-CoV-2 wild type, Omicron BA.1, and Omicron BA.2 stratified by time from last vaccination. Error bars represent 95% confidence interval of means. ns: non-significant, *p<0.05, **p<0.01.

**Figure 2:**
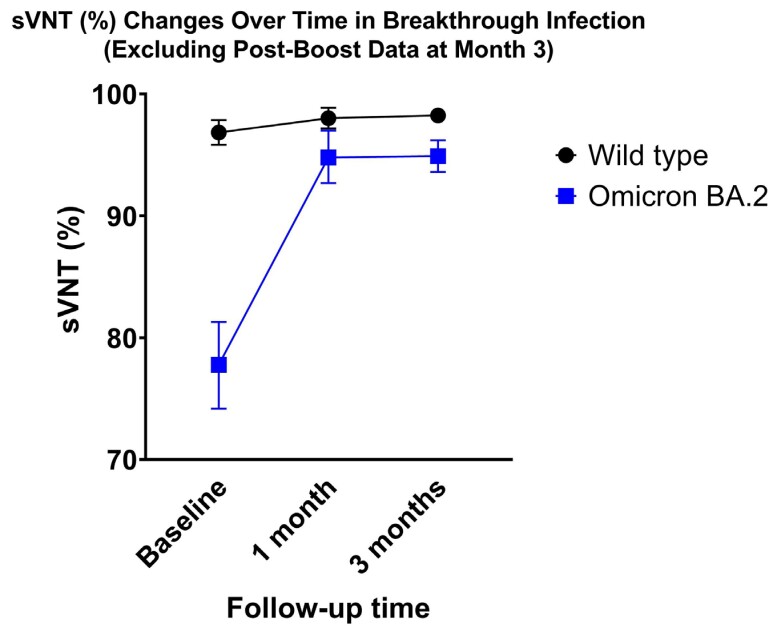
shows the dynamics of sVNT (%) against SARS-CoV-2 wild type and Omicron BA.2. Participants who received booster vaccination before 3 months were excluded.

**Conclusion:**

Our study suggests that, in outpatient setting, neutralizing antibody responses to wild-type and Omicron BA.2 are durable up to three months following natural infection regardless of previous vaccination regimens. Further research is needed to determine the long-term durability of these immune responses and the need for booster vaccination in the context of emerging SARS-CoV-2 variants

**Disclosures:**

**All Authors**: No reported disclosures

